# Gly482Ser PGC-1α Gene Polymorphism and Exercise-Related Oxidative Stress in Amyotrophic Lateral Sclerosis Patients

**DOI:** 10.3389/fncel.2016.00102

**Published:** 2016-04-22

**Authors:** Angelique Pasquinelli, Lucia Chico, Livia Pasquali, Costanza Bisordi, Annalisa Lo Gerfo, Monica Fabbrini, Lucia Petrozzi, Letizia Marconi, Elena Caldarazzo Ienco, Michelangelo Mancuso, Gabriele Siciliano

**Affiliations:** ^1^Departments of Clinical and Experimental Medicine, Neurological Clinic, University of PisaPisa, Italy; ^2^Departments of Surgical, Medical and Molecular Pathology, and Critical Area, University of PisaPisa, Italy

**Keywords:** amyotrophic lateral sclerosis, exercise, oxidative stress, PGC-1α, polymorphisms

## Abstract

The role of exercise in Amyotrophic lateral sclerosis (ALS) pathogenesis is controversial and unclear. Exercise induces a pleiotropic adaptive response in skeletal muscle, largely through the peroxisome proliferator-activated receptor γ coactivator 1α (PGC-1α), a transcriptional coactivator that regulates mitochondrial biogenesis and antioxidant defense mechanisms. It has been suggested that a Gly482Ser substitution in PGC-1α has functional relevance in human disorders and in athletic performance. To test this hypothesis, we examined the genotype distribution of PGC-1α Gly482Ser (1444 G > A) in ALS patients to evaluate whether or not the minor serine-encoding allele 482Ser is involved in oxidative stress responses during physical exercise. We genotyped 197 sporadic ALS patients and 197 healthy controls in order to detect differences in allelic frequencies and genotype distribution between the two groups. A total of 74 ALS patients and 65 controls were then comparatively assessed for plasmatic levels of the oxidative stress biomarkers, advanced oxidation protein products, ferric reducing ability and thiol groups. In addition a subgroup of 35 ALS patients were also assessed for total SOD and catalase plasmatic activity. Finally in 28 ALS patients we evaluated the plasmatic curve of the oxidative stress biomarkers and lactate during an incremental exercise test. No significant differences were observed in the genotype distribution and allelic frequency in ALS patients compared to the controls. We found significant increased advanced oxidation protein products (*p* < 0.001) and significant decreased ferric reducing ability (*p* < 0.001) and thiol groups (*p* < 0.001) in ALS patients compared to controls. When comparing different genotypes of PGC-1α, no relation between Gly482Ser polymorphism and oxidative stress biomarker levels was detected in resting conditions. On the other hand, when considering exercise performance, lactate levels were significantly higher (between *p* < 0.01 and *p* < 0.001) and greater protein oxidative products were found in AA (Ser482Ser) compared to GG (Gly482Gly) and GA (Gly482Ser) ALS patients. Our findings highlight the importance and confirm the involvement of oxidative stress in ALS pathogenesis. Although not associated with 1444 G > A SNP, ALS patients with Gly482Ser allelic variant show increased exercise-related oxidative stress. This thus highlights the possible role of this antioxidant defense transcriptional coactivator in ALS.

## Introduction

Amyotrophic lateral sclerosis (ALS) is the most common neurodegenerative disorder of motor neurons. Loss of pyramidal, spinal, and bulbar motor neurons affecting multiple regions of the body leads to progressive motor dysfunction, disability and death. It is clinically characterized by progressive paralysis and eventual death from respiratory failure within 2–5 years (Wijesekera and Leigh, 2009; D’Amico et al., 2013).

ALS is mainly a sporadic disease (sALS) however approximately 10% of ALS cases are familial (fALS; Ticozzi et al., 2011), due to mutations in several genes, which can play an important role in understanding the pathogenesis of fALS and sporadic ALS (Ingre et al., 2015). The distribution of fALS and sporadic cases also depends on the ancestral origin of the population, since the frequency of ALS-associated genes, as well as environmental factors vary significantly across different populations (Marin et al., 2015).

ALS is a complex and multifactorial disease characterized by the involvement of several pathogenic conditions, including glutamatergic excitotoxicity (Matsumoto et al., 2007), mitochondrial dysfunction (Shi et al., 2010), defects in RNA maturation (Matsumoto et al., 2007) and oxidative stress (Robberecht, 2000). Oxidative stress biomarkers have been investigated in several studies and show high protein carbonyl levels, increased lipid peroxidation and DNA/RNA oxidative modifications, in the nervous and peripheral tissues in both sporadic and fALS ALS patients (Chang et al., 2008; Barber and Shaw, 2009; Kabuta et al., 2015).

ALS occurs in all socio-professional categories with a higher prevalence in athletes and in individuals who practice intense and competitive activities. In fact the association of strenuous physical activity with genetic predisposition and external factors, such as chemicals used in the sanitizing sports grounds, or head injuries suffered by players during games, can increase the risk of developing ALS in some individuals (Scarmeas et al., 2002; Chiò et al., 2005). Conversely moderate activity has been shown to be protective for ALS in mice (Carreras et al., 2010).

Acute exercise is known to induce a transient increase in reactive oxygen species (ROS), as shown by several reports of increased oxidative damage following acute bouts of erobic and anerobic exercise (Fisher-Wellman and Bloomer, 2009). Although the results are somewhat mixed and appear disease dependent, individuals with chronic disease experience an exacerbation in oxidative stress following acute exercise compared to healthy individuals (Fisher-Wellman and Bloomer, 2009). However the relationship between physical activity and oxidative stress is far from linear (Pittaluga et al., 2006), since physical activity is associated with favorable changes in blood pressure, and lipid and lipoprotein profiles, which reduce the risks of several oxidative stress associated diseases. In addition, recent research shows that physical activity contributes significantly to a longer life in good health (Fraile-Bermúdez et al., 2015).

It is still unclear which of these mechanisms are the primary causes or consequences of ALS pathogenesis. Several proteins have been identified that may be involved in the mechanisms of ALS pathogenesis, including the Peroxisome proliferator activated receptor gamma coactivator 1 Alpha (PGC-1α; Qi et al., 2015). PGC-1α is a tissue-specific transcriptional coactivator, encoded by the gene *PPARGC1A/PGC-1*α, located on chromosome 4 (4p15.1), consisting of 13 exons (Arany, 2008).

PGC-1α has an N-terminal domain, containing the sites for the activator proteins, a central region containing the sites for the transcription factors which interact with PGC-1α, and a C-terminal domain containing a binding site for RNA (Monsalve et al., 2000). PGC-1α activates different metabolic pathways in response to nutritional and environmental stimuli such as fasting, cold and physical activity (Handschin et al., 2007a). In physiological conditions, PGC-1α is located in the cytoplasm in an inactive state. During muscle contraction or exercise, it is activated and moves into the nucleus where it promotes the transcription of genes involved in mitochondrial biogenesis, oxidative phosphorylation, skeletal muscle fiber composition remodeling and antioxidant defense (Puigserver and Spiegelman, 2003; Valle et al., 2005; Liang and Ward, 2006).

Studies in mice models of ALS (SOD1 G93A and SOD1 G37R mouse) and Duchenne muscular dystrophy (mdx mouse; Sheng et al., 2012) have shown that increased expression and activity of PGC-1α can improve the clinical manifestations (Handschin et al., 2007b; Liang et al., 2011) by increasing mitochondrial biogenesis and ROS detoxification (Austin and St-Pierre, 2012). Da Cruz et al. (2012) have shown that increased PGC-1α activity in G37R improves muscular endurance, reduces atrophy and improves locomotor activity also in the advanced stages of disease. Liang et al. (2011) showed that PGC-1α expression declines with ALS development in SOD1 G93A mice and that PGC-1α overexpression slows the disease progression also moderately extending the lifespan, without any effect on disease onset. The motor activity was also improved, with a significant decrease in motor neuron cell death and neuromuscular junction damage, thus suggesting that increased PGC-1α expression may be important for ameliorating ALS progression (Liang et al., 2011).

The gene PGC-1α exists in several allelic variants that differ from the common allele in terms of a single nucleotide (Steinbacher et al., 2015). Among single nucleotide polymorphisms (SNPs), the G to A substitution at position 1444 in exon 8 of the PCG-1α gene, results in the substitution of glycine with serine in codon 482 and has been associated with a lower gene expression and reduced PCG-1α protein activity (Prior et al., 2012).

Several studies have suggested that with increased levels of ROS, PCG-1α can bind several transcription factors that induce the transcription of genes coding for antioxidant enzymes, which in turn limit ROS production and oxidative stress (St-Pierre et al., 2003, 2006; Valle et al., 2005; Liang and Ward, 2006). Other studies have investigated whether SNP 1444 G > A could affect the protein activity as a transcriptional coactivator, since the A carriers have low levels of PCG-1α proteins (Liang and Ward, 2006). In fact, this could inhibit the transcription of antioxidant genes and increase oxidative stress. However, there are conflicting results regarding oxidative stress biomarkers in A carriers and A non-carriers. Weng et al. (2010) found different levels of oxidative stress biomarkers between A carriers and A non-carriers, whereas Lai et al. (2008) did not find any such differences.

During exercise, muscle contraction causes PCG-1α protein activation, increases mitochondrial biogenesis, and oxidative capacity and leads to fast-to slow fiber type conversion (Rasbach et al., 2010). In these conditions the PCG-1α protein can bind a transcription factor myocyte enhancer factor 2 (MEF2). The PGC-1α/MEF2 complex then induces a remodeling of the fiber composition of skeletal muscle with greater involvement of oxidative fibers, which confer resistance to fatigue (Handschin et al., 2003). Zhang et al. (2007) showed that the amino acid change Gly482Ser causes an alteration in the binding site for MEF2, thus affecting the formation of the PGC-1α/MEF2 complex and inhibiting the switch from fast to slow muscle fibers with a greater involvement of glycolytic fibers. On the other hand overexpression of the PGC-1α protein increases the proportion of oxidative fibers (Lin et al., 2002), while knock-out models for PGC- 1α show a change from oxidative to glycolytic fibers (Handschin et al., 2007a). It has also been observed that increased levels of PGC-1α are associated with reduced levels of blood lactate (Wende et al., 2007).

The aim of this study was to evaluate whether or not the PCG-1α SNP 1444 G > A (Gly482Ser) could affect oxidative stress markers, both in basal conditions and during an exercise fatiguing test in ALS patients.

## Materials and Methods

### Subjects

A total of 197 sporadic ALS patients (mean age ± standard deviation (SD): 62.7 ± 11.7 years old) and 197 healthy controls (mean age ± SD: 63.4 ± 9.7 years old) were enrolled in this study. Both groups were recruited at the Department of Clinical and Experimental Medicine, Neurological Clinic, University of Pisa (see Table 1).

**Table 1:** **Experimental design**.

SNP Gly482Ser (1444 G > A)	197 sALS	197 healthy controls
Age (years): mean ± SD	62.7 ± 11.7	63.4 ± 9.7
	↓	↓
Oxidative stress markers:	74 sALS	65 healthy controls
AOPP, FRAP and total thiols	62.4 ± 10.8	74 ± 5.5
Age (years): mean ± SD	↓	
Antioxidant activity: total	35 sALS	
Sod and Catalase	61.4 ± 11	
Age (years): mean ± SD	↓	
Oxidative stress markers during	28 sALS	
exercise: AOPP, FRAP, thiols and lactate	62.9 ± 9.8	
Age (years): mean ± SD		

*sALS, sporadic ALS patients; SD, standard deviation*.

Diagnosis of ALS was conducted according to the criteria of El Escorial, at the onset of the disease. ALS patients showed the “upper limbs” and/or “lower limbs” clinical form with mild or moderate functional disability, as assessed by the clinical scale ALS-FRS-R (The ALS Functional Rating Scale, 1996) and respiratory function was satisfactorily documented by an FVC >70% predicted. FALS ALS cases based on a positive family history of the disease were excluded. Control group included subjects without a medical history of neurological disorders (Table 2). All recruited subjects were of Caucasian origin. Both patients and controls gave their informed consent. The study protocol was approved by the Ethics Committee of the Great North West Area of Tuscany. The study was conducted according to Good Clinical Practice (GCP).

**Table 2:** **Demographic and clinical features of sALS patients and controls**.

Features	sALS patients	Controls
Gender: F/M	82/115	99/98
Age (years): mean ± SD	62.7 ± 11.7	63.4 ± 9.7
Age at onset (years): mean ± SD*	60.2 ± 11.8	–
Disease duration (months): mean ± SD*	83.2 ± 42.5	–
ALS-frs*	40.5 ± 7.2	–
Bulbar*	29	–
Upper Limbs*	46	–
Lower Limbs*	61	–

*Clinical data available for 136 sALS patients. ALS-frs, ALS-functional rating scale.

The association study between the polymorphism Gly482Ser (1444 G > A) in the PGC-1α gene and ALS disease, was investigated using PCR-RFLP method in 197 patients (82 F/115 M) and 197 healthy controls (99 F/98 M).

The cellular redox state was assessed on 74 sALS patients (37 F/37 M) and 65 healthy controls (30 F/35 M), by analyzing plasma levels of various oxidative stress biomarkers. In particular we evaluated the advanced oxidation protein products (AOPPs), as a marker of oxidative damage. We used the ferric reducing antioxidant power (FRAP) and total plasmatic thiol groups as markers of non-enzymatic antioxidants. In addition, in 35 patients (19 F/16 M) we evaluated the total activity of superoxide dismutase (SOD) and catalase enzymes, which are antioxidant enzymatic markers.

Plasmatic levels of oxidative stress markers (AOPP, FRAP, thiols and lactic acid) during exercise, were measured in 28 patients with sALS (15 F/13 M), undergoing an incremental exercise test on the forearm muscles.

### Exercise Protocols

**Scheme 1:**
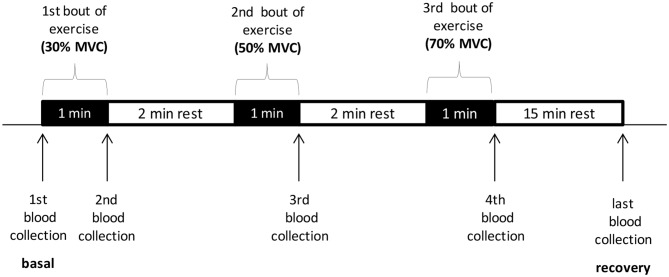
**The exercise test on the forearm muscles conducted with a myometer.** MVC, maximum voluntary contraction.

The exercise test on the forearm muscles was conducted on sALS patients with a myometer connected to a “hand-grip” (Digital Multi-Myometer, MIE Medical Research Ltd., Leeds, UK). The exercise protocol consisted in steps in which the contractile force increased incrementally at 30%, 50%, 70% of the maximum voluntary contraction (MVC), calculated as the mean of three sequential contractions at the myometer, expressed in Newton. Each step includes one minute of intermittent contractions (about 1/s) and two minutes of rest. The venous blood samples for determination of oxidative stress markers were performed at baseline, at the end of each step and 15 min after the end of the exercise (Scheme 1). This type of exercise was mainly erobic at the beginning of the test and it became progressively anerobic, as the force increased, with rapid motor unit recruitment.

### Analytical Procedures

Peripheral venous blood samples were collected both for 1444 G > A (Gly482Ser) single nucleotide polymorphism (SNP) analysis and to monitor the oxidative stress.

#### SNP 1444 G > A Genotyping in PGC-1α Gene

1444 G > A SNP in PGC-1α gene was determined by restriction enzyme analysis using genomic DNA extracted from peripheral blood lymphocytes and the restriction fragment length polymorphism (RFLP) method.

Genomic DNA was isolated from whole blood samples with the QIAamp^®^ Blood Mini Kit (Qiagen).

PCR reaction was performed in a total volume of 25 μl consisting of 1.5 pmol each primer: forward 5′-TGCTACCTGAGAGAGACTTTG-3′ and reverse 5′-CTTTCATCTTCGCTGTCATC-3′, 250 μmol/l dNTPs, Buffer (10X), with Mg^2+^ (25mM), Fast Start Taq [2.5 U] (Roche s.p.a, Italy), and 10 ng of genomic DNA. Reactions were performed in a Perkin Elmer thermal cycler for one cycle at 94°C for 6 min, 30 cycles at 94°C for 30 s, 57°C for 30 s, 72°C for 30 s, and a final extension at 72°C for 7 min.

After digestion with 3 U of MspI (HpaII) restriction enzymes, the amplified fragments were separated by 3.5% metaphor-agarose agarose gel electrophoresis, and the restriction patterns were examinated by ethidium bromide staining and UV light.

#### Evaluation of Oxidative Stress Biomarkers

Peripheral venous blood samples were collected to measure the oxidative stress parameters. Blood samples were immediately centrifuged at 3000 rpm for 10 min and plasma was stored at −80°C until AOPP, FRAP, thiol levels and total SOD and catalase activity were determined. Briefly, AOPPs, a marker of oxidative damage to proteins, were determined by mixing plasma with phosphate buffered saline (PBS), acetic acid and potassium iodide. The absorbance was read spectrophotometrically at 340 nm and compared with a solution of chloramine T dissolved in the same buffer. The data were expressed as nmol/ml of chloramine equivalents (Witko-Sarsat et al., 1996).

In order to measure non-enzymatic antioxidant properties, FRAP was assessed according to Benzie and Strain (1996): the FRAP reagent (sodium-acetate, tripyridyltriazine in hydrochloric acid, and ferric chloride) was pre-warmed to 37°C and mixed with plasma; the absorbance was read after 4 min at 620 nm. A calibration curve was established by substituting the sample with a solution of iron sulfate in hydrochloric acid; the data were expressed in mmol/l.

The content of plasmatic total thiols, which are endogen antioxidants, was estimated by evaluating the sulphydryl groups (-SH) present in the molecules, following the protocol described by Hu (1994).

At the time of determination, 50 μl of plasma were added to 150 μl of Tris—EDTA, 10 μl of 2, 2-dithiobis nitrobenzoic acid (DTNB) and 800 μl of absolute methanol. After incubation at room temperature for 15 min the samples were centrifuged at 3000 g for 10 min. The absorbance of the supernatant was assessed at 412 nm and by subtracting the value of a blank consisting of DTNB. The values were expressed in μmol/l.

The total enzymatic activity of SOD and catalase was detected respectively by superoxide dismutase Assay Kit and catalase assay kit (Cayman, France), respectively, according to the manufacturer’s instructions.

The SOD assay kit uses a tetrazolium salt to detect the superoxide radicals generated by xanthine oxidase and hypoxanthine. Briefly, 10 μl of plasma were mixed with 200 μl of the diluted radical detector, the reaction was initiated by adding 20 μl of diluted xanthine oxidase, and the absorbance was read, after 20 min, at 450 nm. The data were expressed as U/ml of SOD activity. One unit is defined as the amount of enzyme needed to exhibit 50% dismutation of the superoxide radical.

Regarding catalase activity, the kit uses the peroxidatic function of catalase to determine the enzyme activity. Twenty microlitres (20 μl) of plasma were mixed with 100 μl of diluited assay buffer, 30 μl of methanol and 20 μl of diluted hydrogen peroxide. After an incubation for 20 min at room temperature on a shaker, were added 30 μl of catalase purpald and 30 μl of potassium hydroxide. Followed an incubation for 10 min at room temperature and further addition of 10 μl of catalase potassium periodate.

After a final incubation for 5 min, the absorbance of each sample was read at 540 nm. The data were expressed as nmol/min/ml.

The assessment of lactic acid levels, carried out by Kit (Sentinel, Milan-Italy), was conducted in the laboratory of the Department of Clinical Chemistry of the Santa Chiara hospital in Pisa. The kit allows an enzymatic-colorimetric determination of lactate. Lactate is oxidized by lactate oxidase to pyruvate and hydrogen peroxide, which, in presence of peroxidase, reacts with *N-ethyl-N-(2-hydroxy-3-sulfopropyl)-3-methylaniline* forming a compound, whose color intensity is proportional to the concentration of lactate in the examined sample. Plasma (3 μl) were mixed with 300 μl of reagent1, that consists of reagent1a and reagent1b (provided with this kit). After an incubation at 37°C for 10 min, the absorbance of each sample was read at 540 nm. The data were expressed as mg/dl.

#### Data Analysis

To verify whether the allele frequencies were in Hardy-Weinberg equilibrium, and to assess differences in the genotype and allele distributions between groups, we used the Chi-square (χ^2^) analysis using Microsoft Excel. To evaluate any possible alteration in the redox balance in sALS patients and the control group, we used the unpaired two-tailed *t* testing; and data were expressed as mean ± SD. The multi-factor ANOVA test, using the Software StatGraphics, was used to correlate the oxidative stress data with polymorphism G1444A, considering each other biomarker studied as covariates. Only for the ANOVA test, were the values of the considered markers that did not have a normal distribution, prior to performing the assay, were normalized by transforming them into logarithmic data.

The ANOVA test for repeated measures, using the Software SPSS 20 was used to assess any differences in the redox balance during the exercise test. The values of lactic acid, for each exercise step, were normalized compared to the baseline.

A *p* value < 0.05 was considered significant.

## Results

### Analysis of 1444 G > A Polymorphism in PGC-1α Gene

Results obtained from the analysis of the 1444 G > A polymorphism in the PGC-1α gene are shown in Table 3. The distributions of genotypes and allelic frequencies were in agreement with the Hardy-Weinberg equilibrium. We did not observe any difference in the genotype and allelic distribution between the sALS patients and healthy controls.

**Table 3:** **Distribution of the genotypes and allele frequencies of the SNP G1444A in ALS patients and in controls**.

Genotype	sALS *n* (%)	Healthy controls *n* (%)	OR	IC 95%	*p* value
GG (Gly482Gly)	64 (32)	77 (39)	1.00*		
GA (Gly482Ser)	98 (50)	85 (43)			0.15
AA (Ser482Ser)	35 (18)	35 (18)			0.53
A carriers (482Ser)	133 (68)	120 (61)	1.33	0.88; 2.02	0.17
Allel A (482Ser) frequency	0.43	0.39			0.35
Total	197	197			

*Reference value for OR (odds ratio); IC, confidence interval; p value obtained by χ^2^ analysis.

### Evaluation of Oxidative Stress Biomarkers

**Figure 1:**
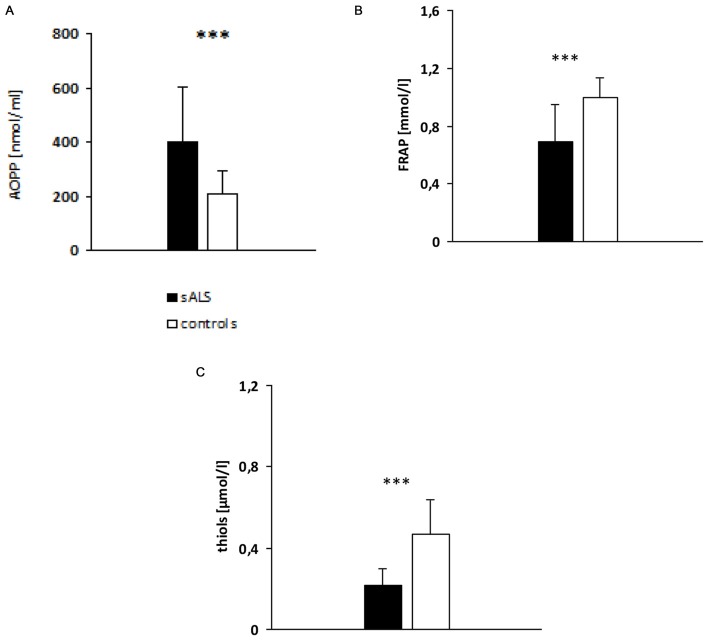
**Evaluation of plasma advanced oxidation protein products (AOPP; A), ferric reducing antioxidant power (FRAP; B) and thiols (C) levels in sALS patients vs. controls.** *p* value obtained by *t*-Student test; ***p < 0.001.

sALS patients had significantly higher AOPP plasma levels (397.91 ± 206.97 nmol/ml; *p* < 0.001) than the healthy controls (Figure 1A). Likewise, patients with sALS had a significantly lower plasma levels of FRAP (Figure 1B) and thiols (Figure 1C) than the controls.

### Correlation Between Oxidative Stress Biomarkers and 1444 G > A SNP in PGC-1α Gene

**Figure 2:**
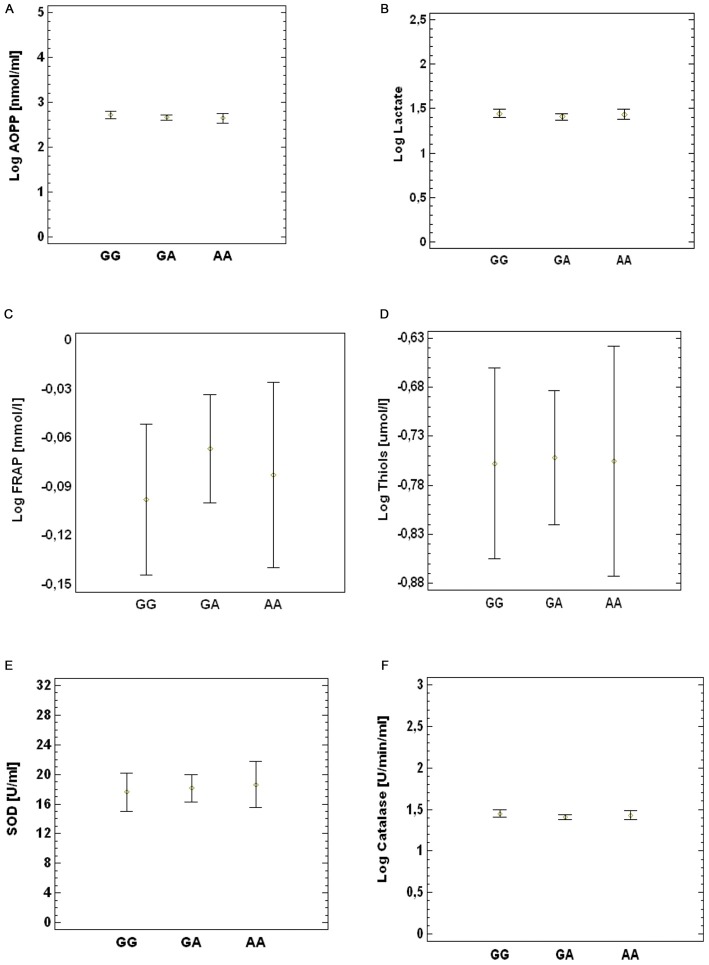
**Association between SNP G1444A and peripheral oxidative damage markers. (A)** AOPP, **(B)** lactate, **(C)** FRAP **(D)**, thiols **(E)**, SOD and **(F)** catalase. *p* value obtained by multi-factor ANOVA test.

The study population (ALS patients and controls) was divided into three groups according to genotypes in GG, GA and AA. We did not observe any difference in the levels of the oxidative stress biomarkers AOPP (Figure 2A), lactate (Figure 2B), FRAP (Figure 2C), thiols (Figure 2D), SOD (Figure 2E) and catalase activity (Figure 2F) between the three groups. The same results were obtained by comparing each of these ALS subgroups with the control group (data not shown).

### Effects of Exercise on Oxidative Stress Biomarkers

**Figure 3:**
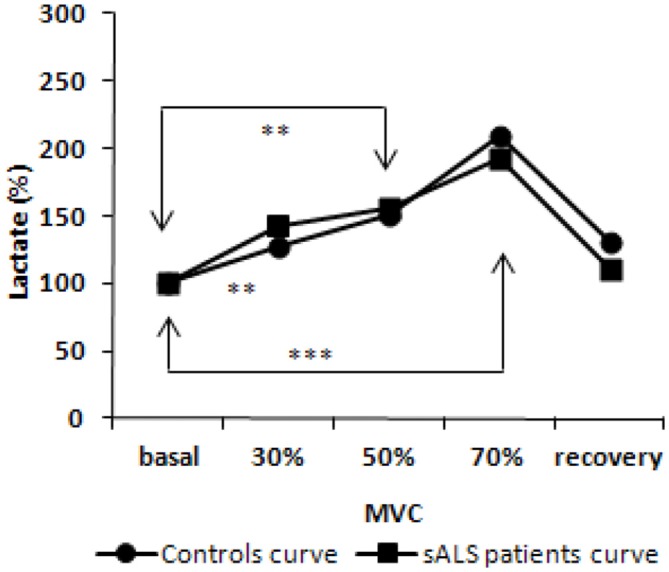
**Lactate curve during incremental workload forearm exercise; normalized values from baseline.** *p* value obtained by ANOVA test for repeated measures [*F*_(2.182, 34.911)_ = 15.5, *p* < 0.001]. **p < 0.01; ***p < 0.001.

Plasma levels of AOPP, FRAP and thiols remained stable during exercise until 15 min after physical activity (data not shown). Variations were observed in lactic acid levels during and after exercise [*F*_(2.182, 34.911)_ = 15.5, *p* < 0.001]. In particular, in sALS patients we found increased lactate levels at 30% (*p* < 0.01), 50% (*p* < 0.01) and 70% (*p* < 0.001) of maximal voluntary contraction (MVC) compared to baseline levels and at 70% (*p* < 0.05) vs. 30% of MVC. At the recovery we observed a decrease in lactate at 50% (*p* < 0.05) and 70% (*p* < 0.001) of MVC although we did not observe any difference comparing the curves of ALS patients and healthy subjects (Figure 3).

### Relationship Between the Variations in Oxidative Stress Biomarkers Levels During Exercise and G1444A SNP in PGC-1α Gene

**Figure 4:**
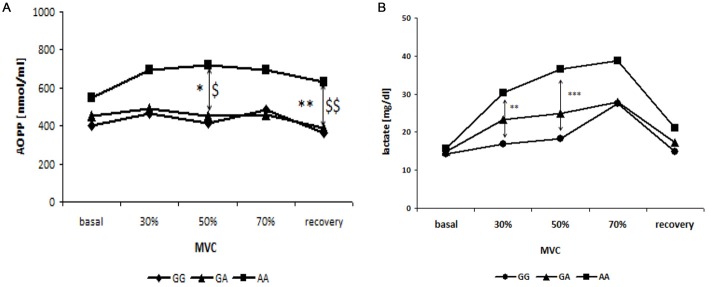
**Relationship between blood curve of AOPP (A) and lactate (B) during incremental workload forearm exercise and SNP G1444A in PCG-1α gene.** *GG vs. AA; ^$^GG vs. GA; ^*, $^*p* < 0.05, ^**, $$^*p* < 0.01, ***p < 0.001.

No changes were observed in the levels of FRAP and thiols in the three groups of patients divided according to genotypes in GG, GA and AA (data not shown). However, changes were observed in AOPP and lactic acid levels. In fact, subjects harboring the 482Ser allele (AA) showed increased AOPP levels during and after exercise than the other two groups (GG and GA genotype) which had stackable levels. This variation was significant at 50% of MVC (*p* < 0.05, GG vs. AA; *p* = 0.05, GA vs. AA) and at recovery (*p* = 0.01, GG vs. AA; *p* < 0.01, GA vs. AA; Figure 4A).

Finally, higher levels of lactic acid were observed during exercise in patients harboring AA genotype, compared to the other two groups. In particular, subjects with AA genotype show significantly increased levels of lactic acid at 30% (*p* < 0.01) and 50% (*p* < 0.001) of the MVC, compared to subjects with the GG genotype (Figure 4B).

## Discussion and Conclusions

*In vitro* studies support the hypothesis that PGC-1α plays a crucial role in protecting the mitochondria from oxidative stress through the reduced accumulation of ROS and reduced apoptotic cell death (Valle et al., 2005; St-Pierre et al., 2006). These data prompted us to evaluate the role of 1444 G > A polymorphism in response to oxidative stress, in basal conditions and during physical activity in sALS patients. ALS is in fact a motor neuronal disorder of a degenerative origin where oxidative stress seems to be one of the pathogenic players and muscle exercise appears to be an important phenotype modulator. We believe that this is the first study investigating PGC-1α SNPs in ALS patients. No differences were found between patients and controls as far as the distribution of 482Ser allele is concerned, suggesting that the PGC-1α Gly482Ser variant is not associated with the disease. This in line with previous data on mice models showing no direct effect of PGC-1α protein expression on ALS onset (Liang et al., 2011; Da Cruz et al., 2012).

To assess the oxidative stress conditions in sALS patients, we have measured plasma levels of the AOPPs, as a marker of oxidative damage to proteins, and the FRAP and total thiol groups, as non-enzymatic antioxidants markers. We also investigated whether Gly482Ser polymorphism affects the levels of the analyzed oxidative stress biomarkers. Patients had significantly higher AOPP levels than the controls, thus confirming the presence of oxidative damage in ALS. These results are in agreement with previous studies indicating that oxidative stress is implicated in this disease (Bogdanov et al., 2000; Simpson et al., 2004; Siciliano et al., 2007; Kabuta et al., 2015). Bogdanov et al. (2000) detected increased levels of 8-OHdG in biological fluids of ALS patients compared to controls. Studies conducted by Simpson et al. (2004) and later confirmed by Kabuta et al. (2015) highlight increased levels of 4-Hydroxynonenal, which is a product of lipid peroxidation, in the spinal cord, motor cortex, cerebrospinal fluid, plasma/serum and urine of sALS patients compared to patients with other neurodegenerative diseases, controls with diseases of non degenerative origin or healthy controls.

Our results also show a reduction in antioxidant defenses, FRAP and thiols, in sALS patients, again confirming previous studies showing decreased antioxidant reserves in ALS. Keizman et al. (2009) showed low serum levels of uric acid, which represents about 60% of the FRAP value, in ALS patients compared to healthy subjects. Other studies have shown decreased total thiols (Baillet et al., 2010) and GSH levels in sALS patients compared to controls (Weiduschat et al., 2014). Overall, our findings of increased AOPPs levels and decreased FRAP and thiol levels in sALS patients seem to reflect an altered redox balance, thus confirming the key role of oxidative stress in ALS pathogenesis.

Under rest conditions, on the other hand, plasma levels of oxidative stress markers (AOPP, lactate, FRAP, total thiol groups, SOD and catalase activity) between ALS patients with GG genotype and A carriers, showed no differences. Thus, the allelic variant A does not seem to exert a negative role in conditions of oxidative stress. It could be speculated that in the PGC-1α protein, the 482 amino acid location, whether or not Gly or Ser, is not a critical domain for interaction with antioxidant gene promoters. However, under rest conditions, the PGC-1α protein is in the cytoplasm in an inactive conformation state and it is activated only in response to stimuli such as fasting, cold, physical activity and high elevation of ROS levels (Park et al., 2014; we did not measure ROS levels in this study).

Physical activity plays a key role in breaking or rebalancing the cellular redox balance (Childs et al., 2001; Iorio, 2003; Waring et al., 2003), and augmenting ROS levels can increase the oxidative damage (Bloomer et al., 2005). Based on this evidence, we investigated possible alterations in the redox balance in response to exercise in a subgroup of sALS patients performing erobic exercise. While lactic acid increased, as expected for this type of exercise, changes in plasma levels of AOPP, FRAP and thiols were not detected during or after muscle exercise in sALS patients. These markers remained roughly constant for up to 15 min after the end of the test. The absence of oxidative stress responses to exercise could nonetheless still be in line with what is known, in healthy subjects, regarding the time relapsing curve of the variations in oxidative stress biomarkers levels after anerobic or erobic exercise. This occurs several hours after the end of the exercise, such as 6, 9, 24 h after exercise, which is a latency considered as compatible with the induction of enzyme machinery behind the oxidative stress markers we considered (Lewin, 2010). For ethical reasons, to avoid excessive discomfort to the patient, we have decided to limit the duration of experimental session and to do not collect samples several hours after the end of the exercise.

PGC-1α regulates the expression of various genes coding for several enzymes involved in fatty acid oxidation. Oxidative phosphorylation and intensive training lead to an increase in PGC-1α levels, which in turn can increase the oxidative capacity in skeletal muscle. A few studies have been conducted to test the possible effect of PGC-1α on athletic performance (Eynon et al., 2010). Lucia et al. (2005) reported an association between exercise and Gly482Ser polymorphism (1444 G > A), the minor A allele being significantly less frequent in athletes than in controls. The 482Ser allele is associated with poor physical capacity, while the Gly482 allele appears to be a genetic factor that increases beneficial muscular endurance (Nishida et al., 2015).

In this work we subdivided sALS patients according to genotype, GG genotype, GA genotype and AA genotype, in order to assess whether the 482Ser allele affects oxidative stress biomarker levels before, during and after exercise. While FRAP and thiol levels did not differ between the three groups, patients harboring the AA genotype showed, during exercise, higher levels of AOPP compared to the other two groups, specifically at 50% of maximal voluntary contraction (MVC) and at recovery after exercise. In addition, subjects with the AA genotype showed significant greater exercise-related lactic acid levels at 30% (GG vs. AA) and at 50% (GG vs. AA) of MVC, compared to subjects harboring GG genotype. The patients with the GA genotype showed intermediate levels of lactic acid, however these data were not statistically significant.

In patients with the AA genotype, the increase in lactate production during the incremental exercise test, may induce an increase in free radicals which, in turn, could damage proteins; in fact we observed an increase in AOPP levels in A carrier subjects. Therefore, although there were no variations in basal oxidative stress biomarkers levels in the 482Ser carriers compared to the Gly482 patients, our results showed that the occurrence of 482Ser allele may contribute to increased oxidative damage during physical activity.

Mechanisms such as the impairment of the MEF2 binding site in PGC-1α, as a result of changes in Gly482Ser amino acid, impede the fast-to-slow fiber type conversion during the endurance response in Gly482Ser carriers (Steinbacher et al., 2015). This can be taken into account to provide a pathogenic substrate to our results. This would then determine a greater involvement of anerobic glycolytic fibers to exercise, with the consequent accumulation of lactic acid, this in turn being one of the responsible factors of increased hydroxyl radical and further oxidative damage. Studies on mRNA expression profiles in targeted tissues would be useful in confirming this. Considering the PGC-1α protective role against muscle atrophy (Da Cruz et al., 2012), we can assume that synthetic molecules able to induce PGC-1α expression, delivered systemically or applied specifically to the muscle, could be used as adjuvant treatments in ALS patients to improve mitochondrial function, reduce atrophy and improve daily physical activity, thus contributing to a better quality of life in this disease.

In conclusion, the results obtained in this work suggest that the Gly482Ser SNP in the PGC-1α gene is related to the lower resistance to exercise-related oxidative stress in ALS. Knowing the genotype of each patient might be helpful in order to design appropriate training protocols aimed at maintaining the functionality of the skeletal muscle for as long as possible.

## Author Contributions

AP and LC wrote the manuscript and performed experiments. ALG and L Petrozzi designed the study. L Pasquali, MM and GS supervised the study. CB, MF, ECI and LM performed clinical evaluation of patients.

## Conflict of Interest Statement

The authors declare that the research was conducted in the absence of any commercial or financial relationships that could be construed as a potential conflict of interest.

## Abbreviations

ALS-FRS-R: Amyotrophic lateral sclerosis functional rating scale
AOPP: advanced oxidation protein products
ALS: amyotrophic lateral sclerosis
DTNB: dithiobis nitrobenzoic acid
fALS: fALS amyotrophic lateral sclerosis
FRAP: ferric reducing antioxidant power
MVC: maximum voluntary contraction
MEF2: myocyte enhancer factor 2
PGC-1α: peroxisome proliferator activated receptor gamma coactivator 1 alpha
PBS: phosphate buffered saline
ROS: reactive oxygen species
RFLP: restriction fragment length polymorphism
SNPs: single nucleotide polymorphisms
SOD: superoxide dismutase
SOD1: superoxide dismutase-1.
